# How was it for you? - obtaining feedback from staff at study sites for the HPS2-thrive trial

**DOI:** 10.1186/1745-6215-16-S2-O52

**Published:** 2015-11-16

**Authors:** Christopher Bray, Elizabeth Wincott, Carol Knott, Martin Landray, Jane Armitage

**Affiliations:** 1 Clinical Trial Service Unit & Epidemiological Studies Unit (CTSU), Nuffield Department of Population Health, University of Oxford, Oxford, UK

## Background

Like other successful Clinical Trials Units (CTUs), CTSU must attract and retain high quality study sites and staff. It is therefore important to understand what study site staff like – and dislike – about working with us, to identify areas for improvement. We conducted a survey to collect feedback from the 245 HPS2-THRIVE sites in the UK, China and Scandinavia.

## Methods

A bespoke questionnaire was provided to the 356 Investigators and Research Coordinators who attended final results meetings. This included sections to characterise the respondent, to allow them to score their experience of different aspects of working on the trial and to provide free-text comments.

## Results

62% of the questionnaires were completed and returned, with equal response rates from Investigators and Research Coordinators. The vast majority (85%) of respondents had been involved in both the recruitment and follow-up phases of HPS2-THRIVE.

90% of the respondents rated their overall experience of working on HPS2-THRIVE as ≥8/10. 90% also said they would be interested in working on another trial coordinated by CTSU, and 93% would recommend working on a future CTSU trial to colleagues. 80% considered their overall experience to be better or much better than other trials they had worked on. Additional questions and analysis provided insights into the underlying factors, including some areas for improvement.

## Conclusion

The survey was easy to manage, with a relatively high response rate. The feedback from study site staff was very informative. We would encourage other CTUs to consider adopting a similar approach.

